# Supplementary material for: Interrater Agreement and Reliability of PERCIST and Visual Assessment When Using 18F-FDG-PET/CT for Response Monitoring of Metastatic Breast Cancer
Source: Diagnostics (Basel). 2020 Nov 24;10(12):1001. doi: 10.3390/diagnostics10121001 (PMC7759893; doi:10.3390/diagnostics10121001)
Supplement: Supplementary file 1 [file diagnostics-10-01001-s001.zip › Table S1.pdf]

Table S1A

Characteristics of primary breast cancer in 37 patients with metastatic breast cancer

**Characteristic**	**No. (%)**
Type of surgery	
Breast conserving	11 (29.7)
Mastectomy	21 (56.8)
No surgery	4 (10.8)
Other	1 (2.7)
Histology	
Ductal carcinoma	26 (70.3)
Lobular carcinoma	3 (8.11)
Carcinoma, unspecified	5 (13.5)
Unknown	3 (8.11)
Size	
≤10 mm	2 (5.41)
11-20 mm	11 (29.7)
21-50 mm	11 (29.7)
≥50 mm	6 (16.2)
Unknown or no surgery	7 (18.9)
Lymph node involvement	
0 or micrometastases	9 (24.3)
1-3	10 (27.0)
4-9	7 (18.9)
≥10	3 (8.11)
Unknown or no surgery	8 (21.6)
Grade	
I	5 (13.5)
II	11 (29.7)
III	9 (24.3)
Unknown	12 (32.4)
ER-status	
Positive	29 (78.4)
Negative	4 (10.8)
Unknown	4 (10.8)
HER2-status	
Positive	9 (24.3)
Normal	18 (48.6)
Unknown	10 (27.0)
Medical treatment	
Neo-adjuvant and adjuvant	9 (24.3)
Adjuvant only	22 (59.5)
No medical treatment	6 (16.2)
	**37 (100)**

ER: Estrogen receptor, HER2: Human Epidermal Growth Receptor 2. Provided with permission and courtesy of Vogsen et al. (PERCIST for longitudinal response monitoring in metastatic breast cancer; under review).

Table S1B

Biomarker profile of metastatic lesion and location of biopsy in 37 patients with metastatic breast cancer

**Characteristic**	**No. (%)**
ER-status	
Positive	30 (81.1)
Negative	6 (16.2)
Unknown	1 (2.7)
HER2-status	
Positive	9 (24.3)
Normal	23 (62.2)
Unknown	5 (13.5)
Location of biopsy*	
Bone	11 (29.7)
Liver	6 (16.2)
Brain	1 (2.7)
Lung	2 (5.4)
Lymph node	9 (24.3)
Breast	2 (5.4)
Skin	2 (5.4)
Other	4 (10.8)
	**37 (100)**

*Location of biopsy from a metastatic lesion, ER: Estrogen receptor, HER2: Human Epidermal Growth Receptor 2. Provided with permission and courtesy of Vogsen et al. (PERCIST for longitudinal response monitoring in metastatic breast cancer; under review).
