# Supplementary material for: Interrater Agreement and Reliability of PERCIST and Visual Assessment When Using 18F-FDG-PET/CT for Response Monitoring of Metastatic Breast Cancer
Source: Diagnostics (Basel). 2020 Nov 24;10(12):1001. doi: 10.3390/diagnostics10121001 (PMC7759893; doi:10.3390/diagnostics10121001)
Supplement: Supplementary file 1 [file diagnostics-10-01001-s001.zip › Table S2.pdf]

Table S2

Association of response/nonresponse assessment using PERCIST by rater

Rater 1 visual	Rater 1 PERCIST		Total (%)
	Response (%)	Nonresponse (%)	
Response (%)	**86 (62.77)**	5 (3.65)	91 (66.42)
Nonresponse (%)	3 (2.19)	**43 (31.39)**	46 (33.58)
Total (%)	89 (64.96)	48 (35.04)	137 (100)

Rater 2 visual	Rater 2 PERCIST		Total (%)
	Response (%)	Nonresponse (%)	
Response (%)	**76 (55.07)**	4 (2.90)	80 (57.97)
Nonresponse (%)	7 (5.07)	**51 (36.96)**	58 (42.03)
Total (%)	83 (60.14)	55 (39.86)	138 (100)

Rater 3 visual	Rater 3 PERCIST		Total (%)
	Response (%)	Nonresponse (%)	
Response (%)	**100 (70.92)**	10 (7.09)	110 (78.01)
Nonresponse (%)	2 (1.42)	**29 (20.57)**	31 (21.99)
Total (%)	102 (72.34)	39 (27.66)	141 (100)
