# Supplementary material for: Interrater Agreement and Reliability of PERCIST and Visual Assessment When Using 18F-FDG-PET/CT for Response Monitoring of Metastatic Breast Cancer
Source: Diagnostics (Basel). 2020 Nov 24;10(12):1001. doi: 10.3390/diagnostics10121001 (PMC7759893; doi:10.3390/diagnostics10121001)
Supplement: Supplementary file 1 [file diagnostics-10-01001-s001.zip › Table S3.pdf]

Table S3

Intrarater response assessment using visual assessment and PERCIST by rater

**Rater 1**	PERCIST				Total (%)
Visual	CMR (%)	PMR (%)	SMD (%)	PMD (%)	
CMR (%)	**17 (12.41)**	0 (0)	0 (0)	0 (0)	17 (12.41)
PMR (%)	1 (0.73)	**39 (28.47)**	9 (6.57)	1 (0.73)	50 (36.50)
SMD (%)	0 (0)	8 (5.84)	**12 (8.76)**	4 (2.92)	24 (17.52)
PMD (%)	0 (0)	1 (0.73)	2 (1.46)	**43 (31.39)**	46 (33.58)
MR (%)	0 (0)	0 (0)	0 (0)	0 (0)	0 (0)
EA (%)	0 (0)	0 (0)	0 (0)	0 (0)	0 (0)
Total (%)	18 (13.14)	48 (35.04)	23 (16.79)	48 (35.04)	137 (100)

**Rater 2**	PERCIST				Total (%)
Visual	CMR (%)	PMR (%)	SMD (%)	PMD (%)	
CMR (%)	**17 (12.32)**	0 (0)	0 (0)	0 (0)	17 (12.32)
PMR (%)	0 (0)	**35 (25.36)**	13 (9.42)	4 (2.90)	52 (37.68)
SMD (%)	0 (0)	0 (0)	**11 (7.97)**	0 (0)	11 (7.97)
PMD (%)	0 (0)	2 (1.45)	2 (1.45)	**49 (35.51)**	53 (38.41)
MR (%)	0 (0)	0 (0)	2 (1.45)	0 (0)	2 (1.45)
EA (%)	0 (0)	0 (0)	1 (0.72)	2 (1.45)	3 (2.17)
Total (%)	17 (12.32)	37 (26.81)	29 (21.01)	55 (39.86)	138 (100)

**Rater 3**	PERCIST				Total (%)
Visual	CMR (%)	PMR (%)	SMD (%)	PMD (%)	
CMR (%)	**14 (9.93)**	0 (0)	0 (0)	0 (0)	14 (9.93)
PMR (%)	1 (0.71)	**45 (31.91)**	11 (7.80)	4 (2.84)	61 (43.26)
SMD (%)	0 (0)	2 (1.42)	**27 (19.15)**	6 (4.26)	35 (25.82)
PMD (%)	0 (0)	1 0.72()	0 (0)	**29 (20.57)**	30 (21.28)
MR (%)	0 (0)	0 (0)	0 (0)	0 (0)	0 (0)
EA (%)	0 (0)	0 (0)	1 (0.71)	0 (0)	1 (0.71)
Total (%)	15 (10.64)	48 (34.04)	39 (27.66)	39 (27.66)	141 (100)

CMR: Complete metabolic response; EA: Equivocal answer; MR: Mixed response; PMD: Progressive metabolic disease; PMR: Partial metabolic response; SMD: Stable metabolic response.
