# Supplementary figures and images for: Interrater Agreement and Reliability of PERCIST and Visual Assessment When Using 18F-FDG-PET/CT for Response Monitoring of Metastatic Breast Cancer
Source: Diagnostics (Basel). 2020 Nov 24;10(12):1001. doi: 10.3390/diagnostics10121001 (PMC7759893; doi:10.3390/diagnostics10121001)

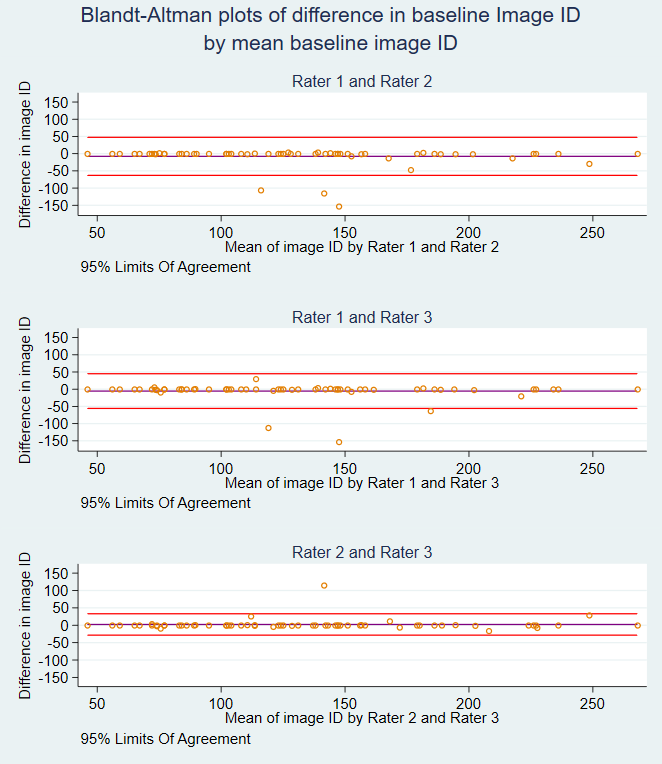

Supplement: Supplementary file 1 [file diagnostics-10-01001-s001.zip › Figure S1.tif]

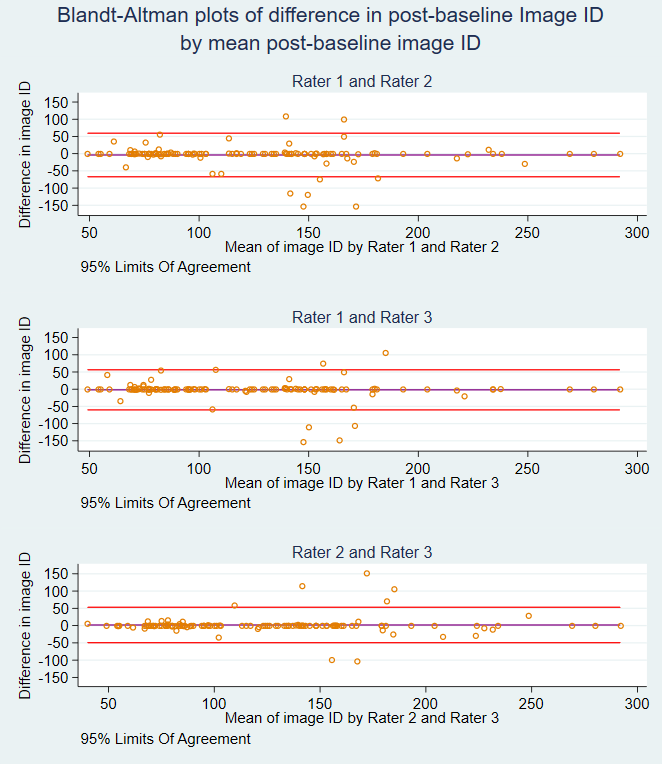

Supplement: Supplementary file 1 [file diagnostics-10-01001-s001.zip › Figure S2.tif]
